# Molecular characterization of pro-metastatic functions of β4-integrin in colorectal cancer

**DOI:** 10.18632/oncotarget.21290

**Published:** 2017-09-27

**Authors:** Wanguang Zhang, Bixiang Zhang, Trung Vu, Guandou Yuan, Binhao Zhang, Xiaoping Chen, Upender Manne, Pran K. Datta

**Affiliations:** ^1^ Birmingham Veterans Affairs Medical Center, Birmingham, AL, USA; ^2^ Division of Hematology and Oncology, Department of Medicine, UAB Comprehensive Cancer Center, University of Alabama at Birmingham, Birmingham, AL, USA; ^3^ Hepatic Surgery Center, Tongji Hospital, Tongji Medical College, Huazhong University of Science and Technology, Wuhan, China; ^4^ Division of Hepatobiliary Surgery, The First Affiliated Hospital of Guangxi Medical University, Nanning, China; ^5^ Department of Pathology, University of Alabama at Birmingham, Birmingham, AL, USA

**Keywords:** colorectal cancer, liver metastasis, affymetrix microarray, β4-integrin, tissue microarray

## Abstract

The β4-integrin subunit has been implicated in development and progression of several epithelial tumor types. However, its role in metastases of colorectal cancer (CRC) remains elusive. To study CRC metastasis, we generated a highly invasive, metastatic cell line MC38-LM10 (LM10) by passaging mouse CRC MC38 cells ten times, using a splenic injection model of liver metastasis. Affymetrix microarray analyses of LM10 and MC38 cell lines and their corresponding liver metastases generated a gene signature for CRC metastasis. This signature shows strong upregulation of β4-integrin in LM10 cells and corresponding metastases. Upregulation of β4-integrin in highly aggressive LM10 cells is associated with increased migration, invasion, and liver metastases. Furthermore, stable knockdown of β4-integrin in human CRC SW620 cells reduces Bcl-2 expression, increases apoptosis, and decreases invasion, tumorigenicity, and liver metastasis, thus resulting in significantly increased survival of mice (hazard ratio = 0.32, 95% confidence interval = 0.15-0.66, P<0.01). Patients with CRC tumors display higher β4-integrin levels in stages 1-4 and significantly lower survival rate. Collectively, β4-integrin plays a critical role in CRC progression, invasion, and metastasis, suggesting that it could be a potential therapeutic target for CRC patients.

## INTRODUCTION

Colorectal cancer (CRC) represents the second most frequent type of cancer-related deaths in the United States [1]. Approximately 15-25% of patients present with liver metastases at the time of primary tumor diagnosis and another 20% of patients will develop liver metastases post-diagnosis [2]. Even with surgery and modern neoadjuvant therapy, most liver metastases will lead to fatalities, as the 5-year survival of patients with distant CRC metastasis is only 12.5% [3]. Thus, understanding the underlying mechanism for the progression of CRC metastasis is essential for the management of this life-threatening disease.

Integrins are transmembrane glycoproteins and are an important family of heterodimeric cell adhesion receptors. They have been shown to be critical in controlling cell-cell and cell-matrix interactions, and regulate a variety of cellular functions crucial to the initiation, progression, and metastasis of solid tumors [4, 5]. α6β4-integrin plays a key role in the formation and stabilization of junctional adhesion complexes called hemidesmosomes [6, 7]. β4-integrin contains a long cytoplasmic domain (1017 amino acid), which does not share homology with other known mammalian integrins [8]. This large and structurally-unique cytoplasmic domain of β4-integrin associates with cytoskeletal elements and is involved in cellular signaling functions [9, 10]. It associates with and enhances the signaling function of multiple receptor tyrosine kinases, including ErbB2 [11], Met [12], and Ron [13]. Once activated, these kinases lead to phosphorylation of the cytoplasmic domain of β4-integrin. Conversely, the α6β4-integrin can promote the phosphorylation of associated tyrosine kinase receptors via Src activation [14]. Studies have shown that β4-integrin can activate Ras and PI3K signaling pathways [5, 15]. Additional studies have demonstrated that higher levels of β4-integrin and changes in its distribution correlate with increased aggressiveness of tumors and poor prognosis [16, 17]. Studies have also demonstrated that β4-integrin enhances cell survival, proliferation, migration, and invasion of various tumors [16, 18, 19]. However, its role in human CRC liver metastasis has not been well documented.

To identify genes that are potentially involved in the promotion of liver metastases of CRC, it is important to acquire an ideal cell line for *in vitro* studies and *in vivo* animal models. In order to generate a highly metastatic cell line MC38-LM10 (LM10), we passaged the parental, less aggressive MC38 cells (a mouse colon cancer derived cell line) ten times using a splenic injection model of metastasis. Following microarray analyses using total RNA from parental MC38, LM10 cells, and corresponding liver metastases, we successfully identified a metastasis signature of CRC through comprehensive gene expression profiling. Interestingly, upregulation of β4-integrin in liver metastases provides evidence of the potential role of β4-integrin in CRC metastasis. Stable knockdown of β4-integrin reduced Bcl-2 expression, increased apoptosis, and decreased invasion, tumorigenicity, and liver metastasis, thus resulting in significantly increased survival of mice. Our observations reveal elevated levels of β4-integrin in CRC patients’ primary tumors and liver metastases, and thus blockade of β4-integrin may represent a therapeutic approach for CRC treatment.

## RESULTS

### Generation of a highly metastatic colon cancer cell line

**Figure 1:**
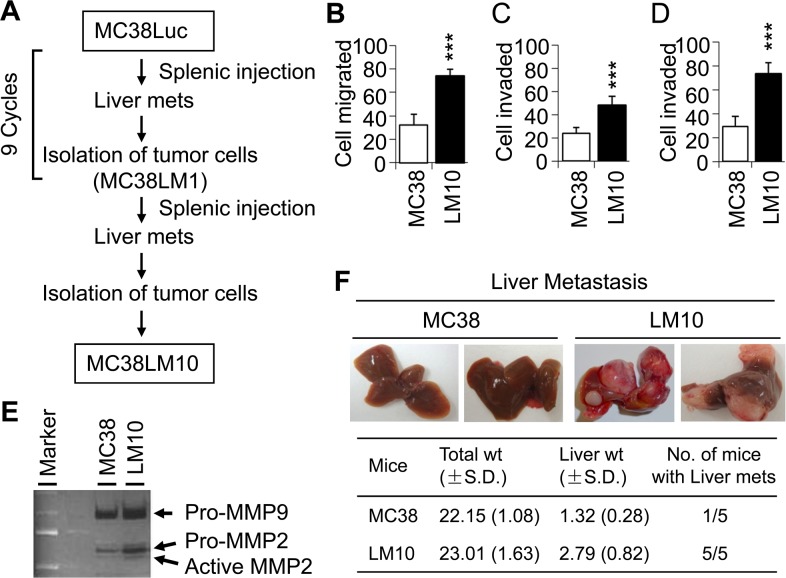
LM10 cells display more aggressive characteristics than parental MC38 cells **(A)** Schematic representation of experimental flow chart, showing the generation of LM10 cells after 10 cycles of splenic injection. **(B)** Migration assay was performed to assess the aggressive characteristics of LM10 cells. Data are presented as mean ±SD from three independent wells. ^***^p<0.001 when compared with control. **(C** and **D)** Cell invasion assay through collagen (C) or matrigel (D) was performed as described in Materials and Methods. Data are presented as the mean ±SD from three independent wells. ^***^p<0.001. **(E)** Gelatinase zymographic analyses of MMP-2 and MMP-9 activity were performed using parental MC38 and LM10 cells. Both MMP-2 and MMP-9 activity was increased in LM10 cells compared to parental MC38 cells. **(F)** MC38 and LM10 cells were injected into spleens of C57BL/6 mice (5 mice in each group). Liver metastasis was assessed after four weeks of splenic injection.

To establish a representative and reproducible animal model to study CRC metastasis, we generated a highly metastatic mouse cell line using MC38 luciferase cells (MC38-Luc). These cells express luciferase and neomycin resistance genes, and were used in a splenic injection model of liver metastasis. MC38-Luc cells were injected into the spleens of C57BL/6 mice and three weeks later, mice were sacrificed, liver metastases were harvested, and the cell line was established by neomycin selection. A highly metastatic MC38-LM10 (named as LM10) cell line was established after 10 cycles of stepwise selection (Figure 1A).

LM10 cellular aggressiveness was tested by *in vitro* migration and invasion assays using Boyden chambers. We found that LM10 cells exhibited a 2.3-fold increase in cell migration compared with MC38 cells (Wilcoxon Rank Sum test, p<0.001) (Figure 1B). In comparison to MC38 cells, LM10 cells displayed an increase in cell invasion by 2.0-fold (through collagen) and by 2.5-fold (through matrigel) (Wilcoxon Rank Sum test, p<0.001) (Figure 1C and 1D, respectively). We subsequently determined MMP activities of LM10 cells using zymography assays and found that LM10 cells showed higher levels of MMP-2 and MMP-9 activity compared to those of MC38 cells (Figure 1E). To test the metastatic potential of LM10 cells, we performed splenic injection. We observed that LM10 cells produced significantly higher levels of liver metastases (4 fold increase, Wilcoxon Rank Sum test, p<0.001) than MC38 cells (Figure 1F). The liver weight of LM10 group is 2.1 times higher than MC38 group (Wilcoxon Rank Sum test, p<0.05). All five mice (100%) in LM10 group developed liver metastases within 4 weeks, whereas one out of five (20%) mice in MC38 control group produced a small liver metastatic focus (less than 0.5 cm). In LM10 group, liver metastases were uniformly distributed throughout the liver parenchyma with a slight predominance to periphery. LM10 cells produced multiple metastatic foci per mouse that were 1 to 1.5 cm in size. Therefore, these results suggest that LM10 cells are highly aggressive and have higher potential to develop liver metastasis.

### Identification of a gene expression profile associated with liver metastasis

**Figure 2:**
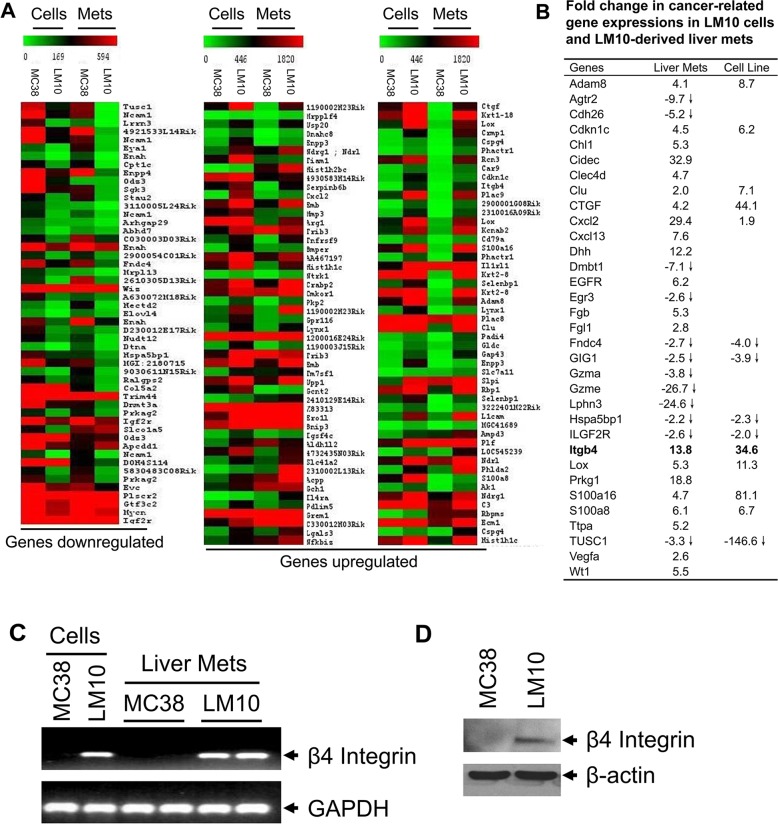
Gene expression profile analyses **(A)** Showing differentially-expressed gene profiles both in LM10 cells or corresponding liver metastasis tissues with at least a 2-fold change (>2 or <2) indicated by colored bars (green: down-regulated and red: up-regulated), when compared to MC38 counterparts. **(B)** Relative fold changes of upregulated or downregulated genes in LM10 cells and corresponding liver metastases compared to parental MC38 cells and corresponding liver metastases, respectively. **(C)** The mRNA expression of β4-integrin was increased in LM10 cells and corresponding liver metastases. The mRNA levels of β4-integrin were detected by RT-PCR. PCR products were analyzed by agarose gel electrophoresis. **(D)** Western blot analysis for protein expression of β4-integrin was performed using lysates prepared from parental MC38 and LM10 cells.

To identify the gene expression signature associated with liver metastasis, we performed affymetrix microarray analyses. Gene expression profiles in MC38 and LM10 cells and their corresponding liver metastases were directly compared to evaluate the gene expression changes associated with invasion and metastasis (Figure 2A). Analyses of gene expression profile differences in LM10 and LM10-liver metastases compared to their MC38 counterparts were performed, after grouping expression profiles according to overall biological processes including development, cell adhesion, apoptosis, signal transduction, angiogenesis, and oncogenesis (Supplementary Excel File 1). Several genes, such as Adam8, Cdkn1c, Chl1, Cidec, Clec4d, Clu, CTGF, Cxcl2, Cxcl13, Dhh, EGFR, Fgb, Fgl1, Itgb4, Lox, Prkg1, S100a8, S100a16, Ttpa, Vegfa, and Wt1 showed 2-30 fold higher expression levels (Figure 2B). Proteins encoded by these genes are involved in angiogenesis, cell proliferation, cell cycle regulation, and apoptosis. We also observed downregulation of several genes, including Agtr2, Cdh26, Dmbt1, Egr3, Fndc4, GIG1, Gzma, Gzme, Lphn3, Hspa5bp1, ILGF2R, and TUSC1 (Figure 2B). The corresponding proteins have been shown to modulate cell adhesion and apoptosis. Interestingly, we found that the β4-integrin gene was upregulated by 13.8 fold in LM10 cells and by 34.6 fold in LM10-liver metastases compared to MC38 cells and MC38-liver metastases, respectively (Figure 2B). We first validated the upregulation of β4-integrin expression in LM10 and LM10-liver metastases by RT-PCR and western blot analyses (Figure 2C and 2D, respectively). RT-PCR analyses showed strong expression of β4-integrin in LM10 cells, but no expression in MC38 cells. In addition, liver metastases generated in two mice from LM10 cells showed strong β4-integrin expression, whereas no expression was detected in liver metastases generated from MC38 cells (Figure 2C). We also observed that the β4-integrin protein expression was upregulated in LM10 cells, but not in MC38 cells (Figure 2D). Therefore, these data suggest that β4-integrin is upregulated in highly metastatic colon cancer cells and may play an important role in the progression of liver metastasis.

### Effect of β4-integrin silencing on cell migration, invasion, and anchorage-independent growth

**Figure 3:**
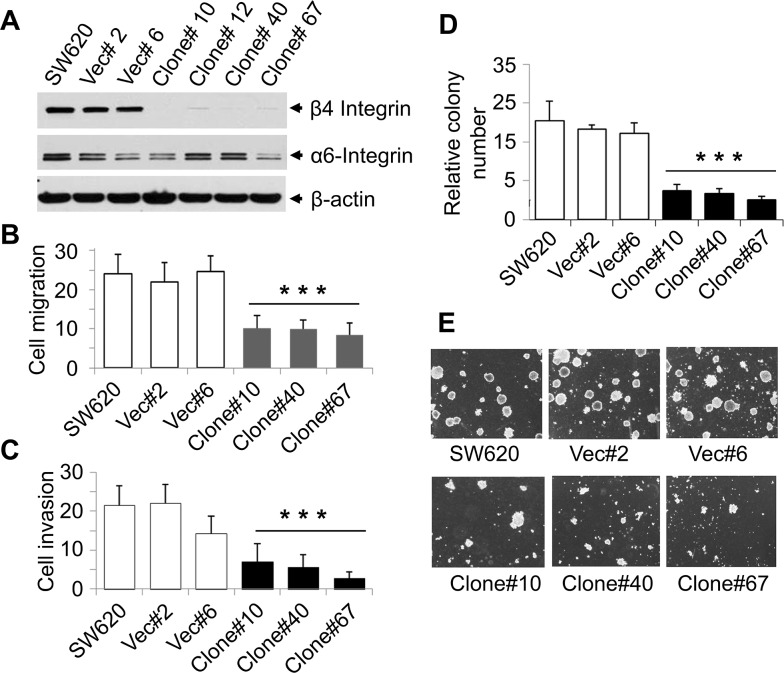
Stable knockdown of β4-integrin in SW620 cells reduced cell migration, invasion, and tumorigenicity **(A)** Stable β4-integrin knockdown clones were generated from SW620 cells, using β4-integrin-pBabe retrovirus and selected in 1μg/ml of puromycin. Expression of β4-integrin in SW620 stable clones was verified by western blotting with β-actin as loading control. **(B)** Migration assay using SW620 cells, two vector control clones, and three β4-integrin knockdown clones was performed as described earlier. Data are presented as mean ±SD from three independent wells. ^***^p<0.001 compared to control. **(C)** Invasion assay through Matrigel was performed using above cells as described in Materials and Methods. Data are presented as the mean ±SD from three independent wells. ^***^p<0.001 compared to control. **(D** and **E)** Decrease in anchorage-independent growth of β4-knockdown clones. Soft agar assays were performed in 35-mm dishes and colonies were allowed to grow for 14 days. Total number of colonies grown on soft agar was counted and shown. Each data point is the average of three values determined from three independent plates. Pictures were taken with a magnification of 200X. These experiments were performed four times in triplicate with similar results.

Upregulation of β4-integrin in a highly tumorigenic colon cancer cell line suggests that β4-integrin may promote CRC progression and metastasis. To determine the pro-oncogenic functions of β4-integrin and to validate the effect of β4-integrin upregulation in human colon carcinoma, we generated stable β4-integrin knockdown SW620 clones using retroviral vectors expressing specific shRNAs (Figure 3A). We have chosen this cell line as it is derived from lymph node metastasis and it expresses high level of β4-integrin (Supplementary Figure 1). We did not observe any significant changes in α6-integrin (a functional partner of β4-integrin) expression in these knockdown clones. We observed that these knockdown clones showed significant reduction in migration as compared to SW620 cells or vector control clones (Ratio of Vec/Clone = 2.47, Wilcoxon Rank Sum test, p<0.001) (Figure 3B). Similarly, we observed that β4-integrin knockdown clones showed significantly reduced invasion through the matrigel barrier (Ratio of Vec/Clone = 3.4, Wilcoxon Rank Sum test, p<0.001) (Figure 3C). These results suggest a potential role of β4-integrin in inducing SW620 cell migration and invasion. The tumorigenic potentials of these stable β4-integrin knockdown clones were verified by anchorage-independent growth assays. β4-integrin knockdown SW620 clones showed significantly reduced colony numbers in soft agarose assays compared with parental cells or vector control clones (Ratio of Vec/Clone = 3.01, Wilcoxon Rank Sum test, p<0.001) (Figures 3D and 3E). These results suggest that β4-integrin contributes to tumorigenicity and invasive ability of CRC cells.

### Effect of the loss of β4-integrin expression on apoptosis in colon cancer cells

**Figure 4:**
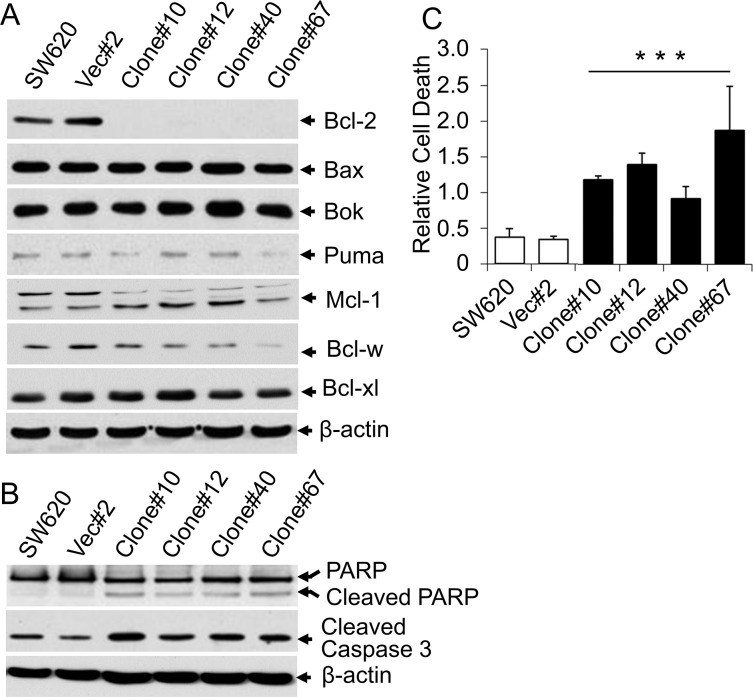
Knockdown of β4-integrin induced apoptosis **(A)** Lysates from SW620 cells, a vector control clone, and stable β4-integrin knockdown clones were subject to immunoblotting with antibodies against Bcl-2, Bcl-xl, Bcl-w, Bax, Bok, Puma, Mcl-1, and β-actin. **(B)** Above cell lysates were also analyzed for expression of cleaved Caspase-3 and PARP. Equal amounts of protein loading were verified by immunoblotting with anti-β-actin antibody (bottom). **(C)** Cell death ELISA assays. Parental SW620 cells, a vector control clone, and stable β4-integrin knockdown clones were processed for cell death apoptosis assay. 10 μl of each clear cell lysate was used for ELISA according to the manufacturer's protocol. Each data point represents the mean ±SD of three individual measurements. ^***^p<0.001 when compared with control.

To gain insight into the mechanism by which β4-integrin induces tumorigenicity of colon cancer cells, we determined whether β4-integrin is involved in regulating cell proliferation and/or apoptosis. We did not observe any change in cell growth between control cells and knockdown clones in cell counting and thymidine incorporation assays (data not shown). We next tested the expression of apoptosis related proteins in the knockdown clones. Analyses of protein lysates from SW620 clones showed strong downregulation of anti-apoptotic Bcl-2 in knockdown clones compared to vector control clone and SW620 cells (Figure 4A). Other anti-apoptotic protein expression (Bcl-w, Bcl-xl, and Mcl-1) and pro-apoptotic protein expression (Bok, Bax, and Puma) were not affected. However, the apoptotic markers (cleaved PARP and cleaved Caspase-3) were increased in all four β4-integrin knockdown clones (Figure 4B). These results suggest an anti-apoptotic role of β4-integrin in SW620 cells. To further confirm the induction of apoptosis in β4-integrin knockdown clones, we performed cell apoptosis assays. All four β4-integrin knockdown clones showed substantial enhancement of cell death (2.5 to 5.5 fold) compared to SW620 or vector control clone (Ratio of Vec/Clone = 0.26, Wilcoxon Rank Sum test, p<0.001) (Figure 4C). These results demonstrate that increased apoptosis, rather than decreased cell proliferation, was responsible for reduction in tumorigenicity of β4-integrin knockdown clones. In addition, β4-integrin inhibits apoptosis by upregulating anti-apoptotic Bcl-2 in SW620 cells.

### Loss of β4-integrin reduces tumorigenicity and metastatic potential of SW620 cells

**Figure 5:**
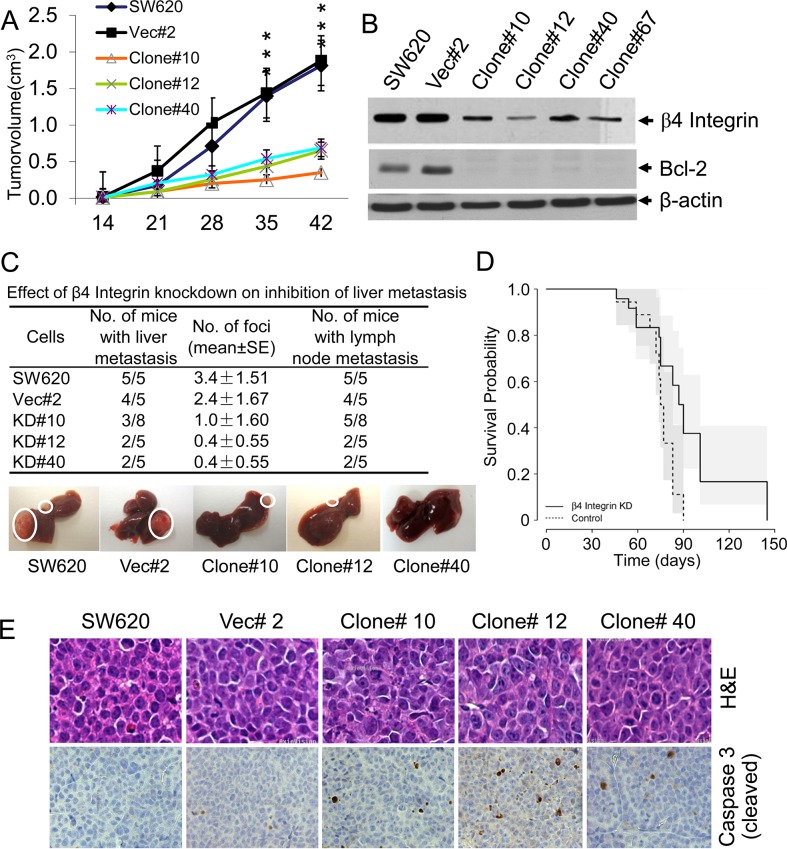
Stable knockdown of β4-integrin showed decreased tumorigenicity and liver metastasis **(A)** Subcutaneous tumors were generated in nude mice by injecting above β4-integrin knockdown clones in parallel with SW620 cells and vector control clone. Tumor volumes were measured and tumor growth curves are presented as the mean volume ±SD of tumors (n=5). ^***^p<0.001. **(B)** Reduced levels of β4-integrin were maintained in subcutaneous tumors with knockdown clones compared to those from parental cells and vector clone. Low levels of β4-integrin were correlated with low levels of Bcl-2. **(C)** SW620 clonal cells were injected into spleens of athymic nude mice (n=5, in each group). Eight weeks after injection, the mice were killed and macroscopic liver metastasis and lymph node metastasis were assessed and are shown by circles in one mouse from each group. Number of liver foci and number of mice with liver and lymph node metastasis are shown. **(D)** The survival of nude mice injected with β4-integrin knockdown (n= 20) or vehicle (n= 18) control cells was analyzed. ^**^p<0.01 when compared with control. **(E)** H&E and activated Caspase-3 immunohistochemistry in liver metastases generated from β4-integrin knockdown/control clones. Paraffin sections were immunostained with a polyclonal antibody which specifically detects activated Caspase-3 and staining signals were detected in cellular cytoplasm.

To determine whether β4-integrin contributes to *in vivo* tumorigenicity, SW620 cells and clones were subcutaneously injected into athymic nude mice. β4-integrin knockdown clones showed significantly reduced growth compared to control groups (Figure 5A). Lower β4-integrin protein expression in subcutaneous tumors was confirmed by western blot analyses. Tumors from the β4-integrin knockdown clones also showed reduced expression of anti-apoptotic Bcl-2 (Figure 5B).

We next investigated the role of β4-integrin in liver metastasis using a splenic injection model and the survival of mice bearing liver tumors derived from SW620 cells, a vector control clone, and three β4-integrin knockdown clones (Figures 5C and 5D). We observed that 100% mice (5 out of 5) injected with SW620 cells and 80% mice (4 out of 5) injected with vector control clone produced liver metastases, whereas 39% (considering all clones) of β4-integrin knockdown clone-injected mice showed liver metastases (Figure 5C). Additionally, 100% mice from SW620 cells and 80% mice from vector control clone produced lymph node metastases, whereas 40-62% mice injected with stable clones produced much smaller lymph node metastases (Figure 5C). These results demonstrate the potential role of β4-integrin in the development of colorectal tumor metastasis. Number of liver foci formation was significantly reduced in mice injected with knockdown clones and these mice also showed much longer survival than control mice injected with SW620 or vector control cells {Hazard for β4-integrin knockdown group is 0.32 times that of the control group (95% CI=0.15 to 0.66, p<0.01)} (Figure 5D). These results suggest that blocking the endogenous β4-integrin expression by shRNA suppresses liver metastasis, and prolongs survival of mice bearing metastatic tumors. H&E staining of above liver tumors revealed similar morphology in liver metastases derived from SW620 and vector control cells, or from stable β4-integrin knockdown clones (Figure 5E). We observed increased staining of Caspase-3 cleaved product in liver metastases derived from β4-integrin knockdown clones compared to SW620 cells or vector control clone. These data suggest that β4-integrin induces liver metastases of SW620 cells by inhibiting apoptosis, thereby supporting *in vitro* results.

### Human colon tumors exhibit high levels of β4-integrin

**Figure 6:**
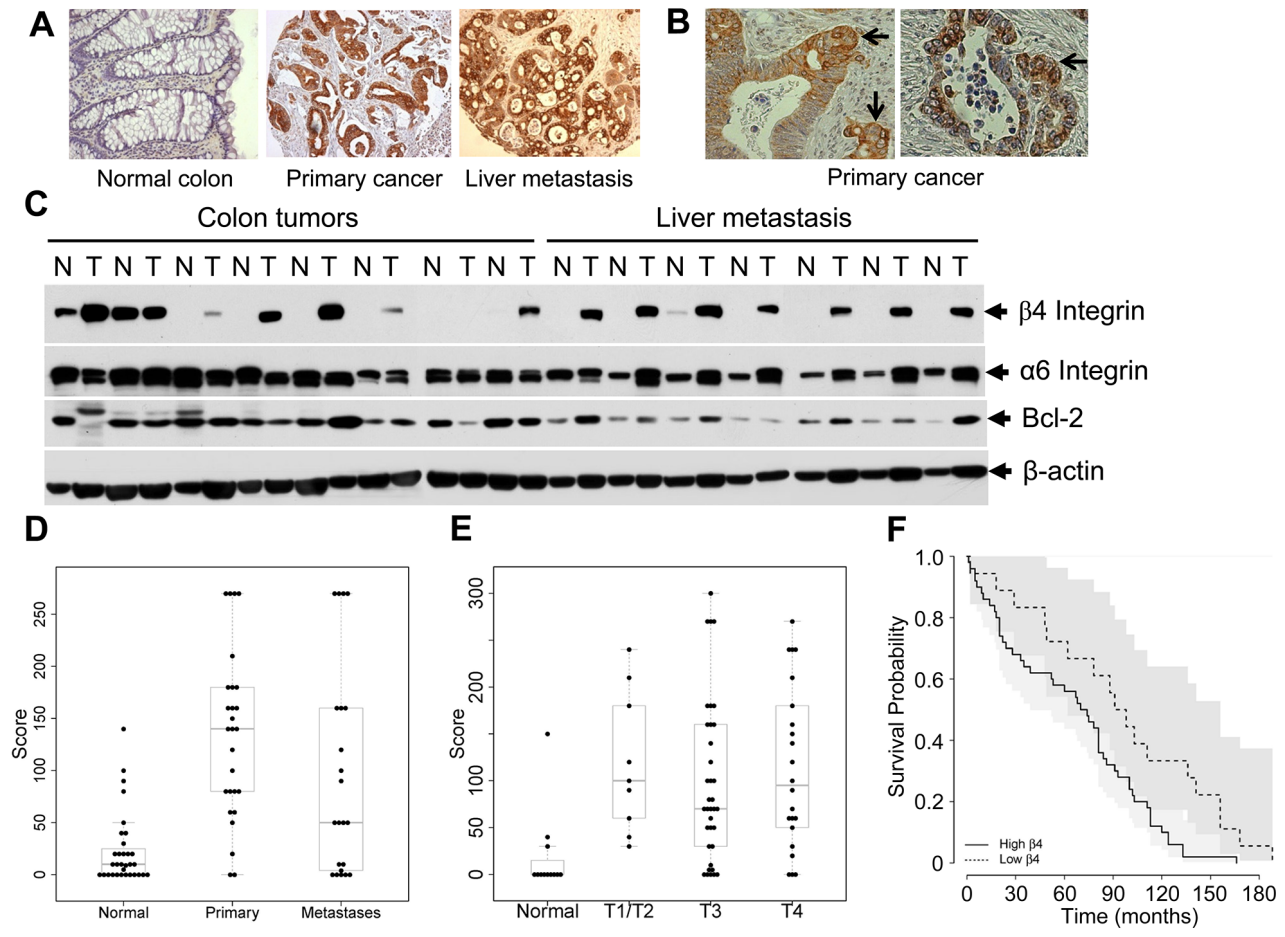
Higher levels of β4-integrin expression in CRC patients were associated with poor survival rate **(A)** Representative β4-integrin immunohistochemical staining of tissues from normal human colon, primary colon cancer, and liver metastasis. A high level of β4-integrin was detected in CRC and liver metastatic tissues (200X). **(B)** At the invasive front of colon carcinoma (indicated by arrows), β4-integrin levels were increased (200X). **(C)** Lysates, from 8 paired normal and primary colon cancer tissues and 7 paired CRC liver metastasis tissues (T) and the patient-matched normal tissues (N), were subjected to immunoblotting with anti-β4-integrin, α6 integrin, Bcl-2, and β-actin antibodies. **(D** and **E)** Both in primary colon tumor and metastasis, β4-integrin expression was upregulated when compared with normal tissues (Chi-square test, p<0.05, D), but no significant differences were observed among different stages (p>0.05, E). **(F)** Analysis of survival (Kaplan-Meier) considering patients with β4-integrin expression. Overall survival of colon cancer patients increases with lower β4-integrin status (p<0.05).

To investigate whether β4-integrin expression is regulated in CRC patients, we performed immunohistochemistry and western blot analyses for β4-integrin expression using patient colorectal tumors and corresponding normal colonic mucosa. We used a CRC tissue microarray (TMA) containing 100 primary colorectal tumors with matched normal tissues, and a metastasis TMA containing 34 matched normal tissue, primary, and metastatic tumors (examples in Figure 6A and 6B). A high level of immunoreactivity for β4-integrin was detected in CRC tumors, predominantly localized in the cytoplasm or membrane. In contrast, very weak immunoreactivity for β4-integrin was observed in normal colon tissues. The relationship between expression levels of β4-integrin and clinicopathologic characteristics is summarized in Supplementary Table 1. No significant relationship was found between β4-integrin expression level and age of patient, tumor site, grade, and depth of invasion. The β4-integrin expression stays higher in primary and metastatic tumors (Figure 6D) and in stages 1-4 (Figure 6E), compared to their corresponding normal controls. We have confirmed this observation by comparing primary tumor β4-integrin expression of 274 colorectal adenocarcinoma (COAD) patients with normal tissue expression from 41 controls using TCGA dataset (http://ulcan.path.uab.edu/index.html, [20]). The β4-integrin mRNA level was increased in all CRC stages compared to normal colon tissue (Supplementary Figure 2). The β4-integrin expression was significantly associated with disease outcome; that is, patients with high β4-integrin expression had shorter survival (Figure 6F, p<0.05) compared to CRC patients with low β4-integrin expression. Remarkably, at the invasive front of colon carcinoma, the β4-integrin levels were increased (Figure 6B). We further confirmed higher carcinoma levels of β4-integrin in a subset of patients by western blot analyses and the data were correlated with immunohistochemistry. As shown in Figure 6C, we analyzed 15 tumor samples with corresponding normal tissues, of which 8 pairs normal and primary tumor, and 7 pairs normal and liver metastatic tissues. All liver metastatic tissues expressed higher β4-integrin levels, whereas corresponding normal tissues didn't express β4-integrin or levels were undetectable. In colorectal primary tumors, 6 of 8 tumors showed higher β4-integrin expression compared to corresponding normal tissues. Taken together, these results demonstrate that high levels of β4-integrin may be associated with human CRC progression and metastasis.

## DISCUSSION

Overall CRC incidence and death rates have steadily declined in the past 30 years, due to the improvement of early detection and/or treatment [3]. However, liver metastases development is still an ominous event in the natural history and progression of CRC. A higher mortality rate of CRC patients is largely due to liver metastasis. Therefore, understanding the mechanisms which underlie human CRC development and progression is important in finding a potential cure for this disease.

In order to identify genes that potentially promote liver metastasis of CRC, it is essential to acquire appropriate cell lines and animal model systems. Few human cell lines metastasize in a splenic injection model. To study CRC metastasis *in vivo* using cell lines, immunocompromised mice are commonly used, but this is not an optimal model system. To circumvent this problem, we have used a mouse cell line and a immunologically-compatible syngeneic mouse model. MC38 cells are not highly invasive and have less potential to produce liver metastases in mice; therefore, we generated a more aggressive and metastatic variant of these cells (LM10) by ten passages of liver metastases using a splenic injection model (Figure 1A). Additionally, to isolate the aggressive version of LM10 cells, these cells were passed through collagen-coated Boyden chamber. Cells which invaded through the collagen layer were trypsin digested and harvested. MC38 cells’ metastatic potential was then compared to that of LM10 cells using a splenic injection model. We observed very similar metastatic potentials of these two cell types (data not shown). Therefore to our knowledge, we are the first to establish a highly invasive, metastatic cell line LM10, thereby providing an appropriate model to identify the metastasis promoting effects of β4-integrin in CRC. We found that both MMP-2 and MMP-9, which are important for promoting cell migration and invasion (Figure 1B, 1C, and 1D), were enhanced in highly metastatic LM10 cells compared to parental MC38 cells. This result is opposite to one report showing that anti-β4-integrin antibodies increase the MMP-2 expression in the colon adenocarcinoma cell line LoVo [21]. However, another study on ovarian cancer showed that β4-integrin depletion reduced the expression of MMP-2 and MMP-9, consistent with our data [22]. We believe that β4-integrin may have different MMP regulation for CRC derived from different genetic backgrounds or stages.

The β4-integrin expression is maintained and often upregulated in various types of invasive and metastatic tumors, and is correlated with poor prognosis [16, 18, 19]. In CRC, the expression of β4-subunit was reported to be reduced/lost in some studies [23, 24], and maintained or heterogeneously increased in other studies [25, 26]. However, no direct correlation was shown between β4-integrin expression and CRC progression and metastasis to the liver. In this study, we show that β4-integrin expression is associated with human CRC progression and metastasis. We first examined the mRNA levels in MC38 and LM10 cells and in their corresponding liver metastases by gene microarray (Figure 2A, 2B). The β4-integrin was highly expressed in LM10 cells compared to parental MC38 cells in both mRNA (Figure 2C) and protein (Figure 2D) levels. We also noticed that there is an upregulation (12.2 fold) of Desert Hedgehog (Dhh), one of the major ligands of Hedgehog signaling, in LM10-liver metastases, compared to MC38 counterparts (Figure 2B). Activation of Hedgehog signaling is reported to be associated with increased metastasis in CRC [27]. A recent study also showed that β4-integrin expression is upregulated by Hedgehog signaling pathway [28]. These results suggest that β4-integrin is upregulated as a result of increased Hedgehog signaling activation in LM10-liver metastases.

Regulation of cell apoptosis is pivotal for cancer progression and metastasis. Apoptosis in tumors or in tumor cell lines could be induced by downregulation of anti-apoptotic proteins, upregulation of pro-apoptotic proteins, or regulation of both. The anti-apoptotic (Bcl-2, Bcl-w, Bcl-xL, and Mcl1) and pro-apoptotic (Bax, Bad, Bok, Bak) Bcl-2 family proteins can affect tumor metastasis through regulation of the mitochondrial apoptotic pathway. Interestingly, in our present study, we provide evidence that β4-integrin knockdown significantly down-regulated Bcl-2 in SW620 cells *in vitro* (Figure 4A) and *in vivo* (Figure 5B) resulting in cleavage of Caspase-3 and PARP (Figure 4B). In addition, Bcl-2 protein expression is positively correlated with β4-integrin in human CRC and liver metastases (Figure 6C). Bcl-w was marginally downregulated when β4-integrin was knocked down in SW620 cells, but other Bcl-2 family proteins did not show obvious changes (Figure 4A). The β4-integrin can interact with epidermal growth factor receptor (EGFR) and promote AKT signaling pathway [29]. Bcl-2 was identified as a target of AKT signaling pathway [30]. As EGFR was also found to be upregulated in LM10-liver metastases, it is possible that β4-integrin/EGFR/AKT axis induces the expression of Bcl-2 through AKT signaling pathway. A second possibility is that α6β4-integrin can activate IGF-1R signaling, which then induces Bcl-2 expression. The α6β4-integrin forms a ternary complex, α6β4-IGF1-IGF1R, which activates IGF-1R signaling and induces Bcl-2 expression [31]. These results provide a potential mechanism by which β4-integrin promotes CRC tumorigenesis and liver metastasis by upregulating the anti-apoptotic protein Bcl-2.

There is no significant difference in β4-integrin expression between primary and metastatic colon cancer (Figure 6D and 6E). An explanation could be that metastatic CRC cells will go through MET at sites of metastases. As β4-integrin also has role in EMT [32], early β4-integrin upregulation in the primary tumor may contribute to metastasis. However, in the secondary tumor, there is no further increase in its expression which may be important for the MET process. Our results show that β4-integrin is upregulated in both protein (Figure 6D) and mRNA levels (Supplementary Figure 2) in all stages of CRC tissue compared to normal tissue. Loss of β4-integrin also reduces tumorigenicity of SW620 cells (Figure 5A). Therefore, it is evident that β4-integrin has roles in both primary colon cancer and metastasis.

Several integrins have been considered as therapeutic targets in cancer treatment. Function-blocking monoclonal antibodies have been developed and show efficacy in preclinical studies [33]. For example, Volociximab, a function-blocking monoclonal antibody against integrin α5β1, has been tested in a phase I trial in patients with advanced solid malignancies. β4-integrin specific antibodies, ASC-8 and ASC-3, have been developed [34], which target different β4-integrin epitopes and have been shown to significantly decrease human keratinocytes migration rate. Based on these studies, new monoclonal β4-integrin antibodies and inhibitors could be developed to block β4-integrin pro-oncogenic functions, including migration, invasion, and metastasis, in preclinical colon cancer models.

In conclusion, we have developed the LM10 cells from mouse colon adenocarcinoma MC38 cells, which produce 100% liver metastasis within three weeks. Microarray analyses show that LM10 cells have significantly higher levels of β4-integrin and activated matrix metalloproteinases, MMP-2 and MMP-9. Knockdown of β4-integrin in human CRC cells reduces liver metastasis and prolongs overall survival of mice bearing liver metastases. This is supported by the fact that patients with higher β4-integrin expression have poorer overall survival. Taken together, our results demonstrate that the β4-integrin is associated with human CRC liver metastasis, and thus, β4-integrin may be a potential target for the development of novel CRC therapeutics.

## MATERIALS AND METHODS

### Cell cultures and animals

MC38 (C57BL/6 mouse colon adenocarcinoma-derived) and SW620 (human colon cancer cells derived from lymph node metastasis) cell lines were cultured as previously described [35]. Female C57BL/6 mice (6-8 weeks old) and athymic nude mice (5-6 weeks old) were used for experiments. Protocols for animal breeding, housing, and handling were approved by the UAB Animal Care and Use Committee (IACUC).

### Reagents and antibodies

Transfection reagents were purchased from Invitrogen (Carlsbad, CA). Puromycin was obtained from Sigma (St. Louis, MO). Antibodies were purchased as follows: anti-human β4 and α6 integrins from Abcam (Cambridge, MA); anti-mouse β4-integrin from R&D (Minneapolis, MN); anti-PARP, anti-Caspase-3, anti-Bcl-2 antibodies, and anti-mouse and anti-rabbit secondary antibodies from Santa Cruz Biotechnology (Santa Cruz, CA); anti-Bcl and -xL, anti-Bax and anti-Puma from Cell Signaling (Danvers, MA). Anti-β4-integrin (450-11A) antibody for immunohistochemistry was a kind gift from Dr. Rita Falcioni (Regina Elena Cancer Institute, Italy).

### Generation of β4-integrin knockdown stable clones

To generate β4-integrin knockdown stable clones, SW620 cells were transfected with pBabe-retro-β4-integrin shRNA or scramble RNA plasmids (Addgene, Cambridge, MA). After transfection, cells were selected with 1 μg/ml puromycin for 10 days, and then single clones were isolated.

### RT-PCR analyses

Isolation of total RNA from cells and RT-PCR analyses were performed as previously described [36]. Briefly, 2 μg of total RNA was reverse transcribed using mouse β4-integrin forward primer: 5′-cctccctcctatctgggaaga-3′ and reverse primer: 5′-atatctccattgggcctcct-3′. PCR amplification was carried out using 2 μl of above RT reaction mixture, 1 × PCR buffer, 1.5 mM MgCl2, 2U Taq DNA polymerase, and 10 nM of each primer in a 25 μl reaction volume. The RT-PCR products were separated and visualized on a 1.5% agarose gel.

### *In vivo* selection of highly metastatic colon cancer cells

Liver metastases were generated by splenic injection as described [35]. Briefly, 1×10^5^ MC38 cells in 100 μl PBS were injected into spleens of C57BL/6 mice. Five minutes after injection, spleens were removed. Three weeks later, liver metastases were obtained. Individual cells were aseptically harvested from a portion of metastatic liver tumors by collagenase digestion. Cells were selected with G418 (400 μg/ml) for at least one week. We then repeated the splenic injection procedure for nine more cycles to select highly invasive, metastatic MC38-LM10 cells, and hereafter these cells were referred to as LM10 cells.

### Microarray analyses

To characterize gene expression profiles in highly metastatic cells, total RNA was isolated from MC38 cells, LM10 cells, and corresponding liver metastases generated in C57BL/6 mice using these cell lines via splenic injection. Microarray analyses were performed using GeneChip 430 Mouse Exon 1.0 ST Array from Affymetrix, which contains 30 arrays for analyzing 28,853 mouse genes. Signal intensity was detected according to the supplier's instructions.

### Western blot analyses

Cell lysate preparation and western blot analyses were performed as described [37]. In summary, cells were solubilized in mammalian lysis buffer, briefly sonicated, and centrifuged at 14,000 rpm for 15 min at 4°C. Equal lysate amounts were resolved and probed with specific primary antibodies. Specific protein bands were visualized by enhanced chemoluminescence.

### Cell migration and invasion assays

Cell migration and invasion assays were performed as previously described [38]. Briefly, for migration assays, 3×10^4^ cells were seeded into the upper chamber of 8-μm pore transwells. For invasion through either collagen layer or matrigel barrier, 3×10^4^ cells in DMEM containing 0.2% BSA were added to the upper chamber of each well. Medium containing 5% fetal bovine serum was added to the lower chamber. Cells were allowed to migrate through transwell membranes for 5 h for migration assay, for 12 h through collagen layer, and for 21 h through matrigel barrier. In the end, migrated or invaded cells were fixed in 4% paraformaldehyde, stained with crystal violet, and then counted from 5 random fields and averaged. Each experiment was repeated three times with similar results.

### Gelatin zymography

MC38 and LM10 cells were cultured in 12-well plates, serum starved overnight, and then supernatant medium was collected. MMP-2 and MMP-9 activity was measured by in-gel gelatin zymography assay as described [39].

### Soft agarose assays

Anchorage-independent growth on soft agarose was performed according to the method as we described [35]. Briefly, 2.5×10^4^ cells from each pool of parental SW620, vector control clones, and SW620 β4-integrin knockdown clones were suspended in 1 ml of 0.4% sea plaque agarose containing 10% FBS and then plated on the top of 1 ml of semisolid 0.8% agarose in 35-mm plates. Two weeks after plating, colonies grown on soft agarose were counted and pictures of colonies shown.

### *In vivo* tumorigenicity assay

2×10^6^ cells from each pool of parental SW620, vector control clones, and β4-integrin knockdown clones were injected subcutaneously behind the anterior forelimb of Balb/c nude mice (n=5, in each group) as we described [37]. Mice were monitored every week for tumor growth, and tumor volumes were measured using the following equation: V=LxW^2^x0.5, where L is length and W is width of a tumor. Tumor growth curves were plotted as the mean volume ± S.D. of tumors of five mice from each group.

### Survival assays

Survival assay was performed as we described [35]. Briefly, 5×10^6^ cells from each pool of parental SW620, vector control clones, and β4-integrin knockdown clones were used for splenic injection into athymic nude mice as described above. Mice were allowed to live and were euthanized when evidence of advanced bulky disease was visible. Day of death was considered the day of death for survival evaluations. The mean survival time for each group of mice was determined and survival was evaluated by log-rank test.

### Immuohistochemical analyses

A tissue microarray (TMA) containing colorectal carcinoma (CRC) specimens in triplicate with control colonic mucosa from 100 patients (53 females and 47 males; mean age = 64 years) was used for immunohistochemistry. CRC cases were taken from surgical pathology archive, with follow-up ranging from 1 to 168 months. Using TNM staging system, 9 tumors were classified as Stage I, 42 as Stage II, 40 as Stage III, and 10 as Stage IV. In addition, a TMA containing 34 cases of colorectal primary carcinomas matched with liver metastases and normal colonic mucosa was used. Study protocols and procedures were approved by the Institutional Review Boards (IRB) at the UAB Medical Center. Tumor deparaffinized and rehydrated sections were pretreated by microwaving in 1mM citrate buffer (pH 6.0), followed by an overnight incubation with the anti-β4-integrin (R&D, clone 450-11A). Membrane and cytoplasmic expression in neoplastic cells was scored by intensity from 0 to 3, according to the following criteria: 0, no expression; 1+, weak expression; 2+, moderate expression; and 3+, strong expression. Percentage of stained tumor cells was recorded, and results are scored by multiplying the percentage of stained cells (P) with the intensity (I). Formula: Q = P × I; Maximum = 300.

### Statistical analysis

Student's t-test or Mann-Whitney test for non-normally distributed data was used to determine the statistical differences among groups analyzed. Calculations were performed using PRISM 5.0 software for Macintosh (GraphPad Software, San Diego, CA). *P*<0.05 was considered statistically significant.

## SUPPLEMENTARY MATERIALS FIGURES AND TABLES




