# Supplementary material for: Molecular characterization of pro-metastatic functions of β4-integrin in colorectal cancer
Source: Oncotarget. 2017 Sep 27;8(54):92333–45. doi: 10.18632/oncotarget.21290 (PMC5696185; doi:10.18632/oncotarget.21290)
Supplement: Supplementary file 1 [file oncotarget-08-92333-s001.pdf]

Molecular characterization of pro-metastatic functions of β4-integrin in colorectal cancer

SUPPLEMENTARY MATERIALS

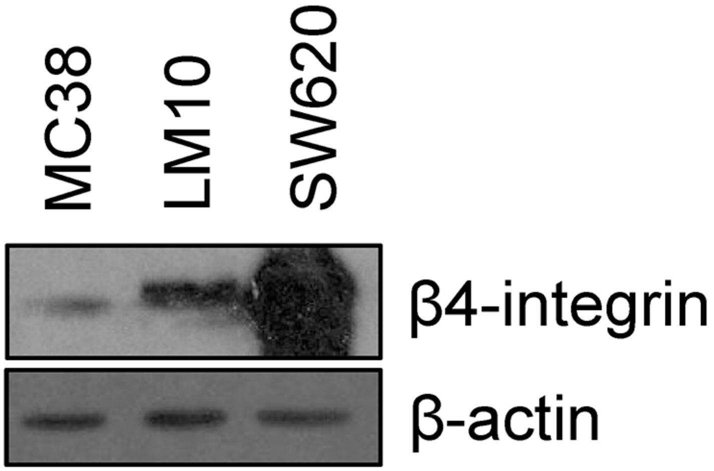

Supplementary Figure 1: Protein expression levels of β4-integrin in MC38, LM10, SW620 cells were analyzed by western blot analyses using antibodies against both human and mouse β4-integrin.

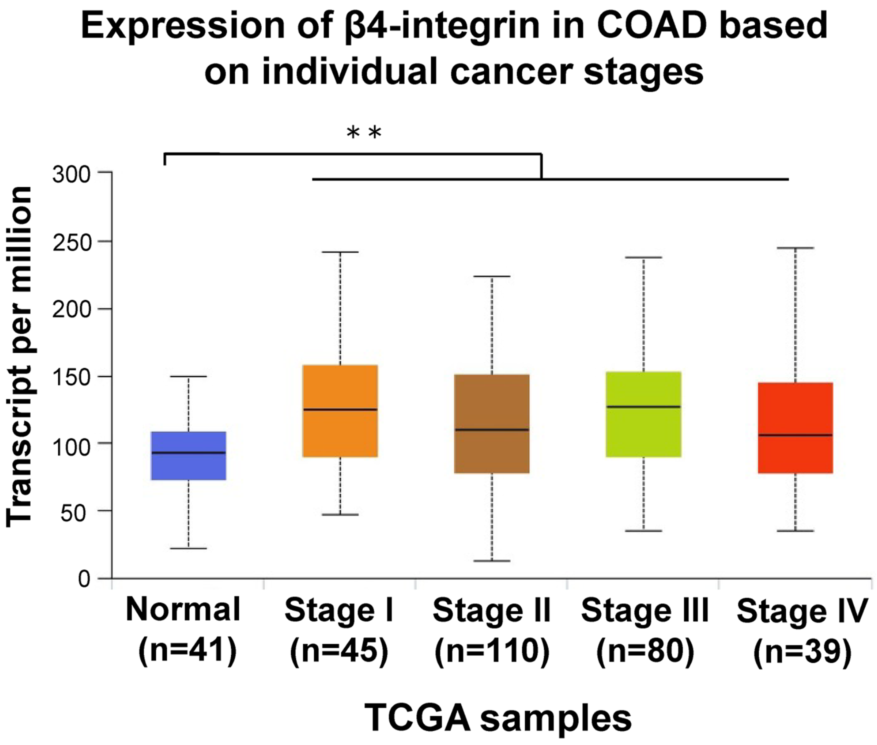

Supplementary Figure 2: TCGA data of 274 colorectal adenocarcinoma (COAD) patients and 41 controls were downloaded from UALCAN: http://ualcan.path.uab.edu/index.html and presented in a box plot.

Supplementary Excel File 1: Gene microarray data.

See Supplementary File 1

Supplementary Table 1: Clinical and pathologic characteristics for high and low β4-integrin expression in human CRC samples

	N	High N=50	Low N=18	Combined N=68	P-value
Age, (*y*, mean ±SD)	67	63.3±15.7	65.0±14.1	63.8±15.2	0.939[1]
Sex	67				0.42[2]
F		51.0% (25)	38.9% (7)	47.8% (32)	
M		49.0% (24)	61.1% (11)	52.2% (35)	
Site	62				0.046[2]
Right		55.6% (25)	29.4% (5)	48.4% (30)	
Left		24.4% (11)	58.8% (10)	33.9% (21)	
Rectum		20.0% (9)	11.8% (2)	17.7% (11)	
Tumor size (cm)	62	5.63±2.71	4.94±1.92	5.45±2.53	0.273[1]
Tumor size cat.	62				0.147[2]
< 5cm		39.1% (18)	62.5% (10)	45.2% (28)	
≥ 5cm		60.9% (28)	37.5% (6)	54.8% (34)	
Grade	68				0.315[2]
1		14.0% (7)	22.2% (4)	16.2% (11)	
2		70.0% (35)	50.0% (9)	64.7% (44)	
3		16.0% (8)	27.8% (5)	19.1% (13)	
Advancing edge	66				0.576[2]
Infiltrative		55.1% (27)	64.7% (11)	57.6% (38)	
Pushing		44.9% (22)	35.35 (6)	42.4% (28)	
Mesenteric deposits	65				1[2]
0		77.1% (37)	76.5% (13)	76.9% (50)	
1		22.9% (11)	23.5% (4)	23.1% (15)	
Vascular invasion	66				0.124[2]
Absent		75.5% (37)	52.9% (9)	69.7% (46)	
Present		24.5% (12)	47.1% (8)	30.3% (20)	
Perineural invasion	65				0.278[2]
0		95.8% (46)	88.2% (15)	93.8% (61)	
1		4.2% (2)	11.8% (2)	6.2% (4)	
Crohn's-like reaction	65				0.778[2]
0		54.2% (26)	47.1% (8)	52.3% (34)	
1		45.8% (22)	52.9% (9)	47.7% (31)	

N is the number of non-missing values. Numbers after percentages are frequencies. Tests used: [1]Wilcoxon test;[2]Fisher's Exact test
